# Increased risk of sleep-related movement disorder in patients with *Helicobacter pylori* infection: A nationwide population-based study

**DOI:** 10.3389/fneur.2022.953821

**Published:** 2022-10-10

**Authors:** Yueh-Feng Sung, Jiu-Haw Yin, Kuang-Heng Lee, Chia-Lin Tsai, Yu-Kai Lin, Shao-Yuan Chen, Chi-Hsiang Chung, Wu-Chien Chien, Jiunn-Tay Lee, Chung-Hsing Chou

**Affiliations:** ^1^Department of Neurology, Tri-Service General Hospital, National Defense Medical Center, Taipei, Taiwan; ^2^Department of Internal Medicine, Taipei Veterans General Hospital, Hsinchu, Taiwan; ^3^Graduate Institute of Medical Sciences, National Defense Medical Center, Taipei, Taiwan; ^4^Department of Neurology, Cardinal Tien Hospital, New Taipei City, Taiwan; ^5^Department of Hyperbaric Medicine, Cardinal Tien Hospital, New Taipei City, Taiwan; ^6^School of Medicine, Fu-Jen Catholic University, New Taipei City, Taiwan; ^7^Taiwanese Injury Prevention and Safety Promotion Association, Taipei, Taiwan; ^8^School of Public Health, National Defense Medical Center, Taipei, Taiwan; ^9^Department of Medical Research, Tri-Service General Hospital, National Defense Medical Center, Taipei, Taiwan

**Keywords:** sleep-related movement disorders, restless legs syndrome, periodic limb movement disorder, *Helicobacter pylori*, movement disorder

## Abstract

**Background and purpose:**

Evidence increasingly suggests that *Helicobacter pylori* infection (HPI) is associated with movement disorders such as Parkinson's disease (PD). However, the relationship between HPI and sleep-related movement disorders (SRMD) remains unknown. This nationwide population-based study tried to demonstrate whether patients with HPI have a higher risk of developing SRMD in a general adult population.

**Methods:**

The study cohort enrolled 9,393 patients who were initially diagnosed with HPI between 2000 and 2013. Notably, 37,572 age- and sex-matched controls without prior HPI were selected as the reference. A Cox proportional hazard regression analysis was performed for multivariate adjustment.

**Results:**

Patients with HPI had a higher risk of developing SRMD (adjusted hazard ratio [HR] = 2.18, 95% confidence interval [CI] = 1.26–3.82, *p* < 0.01). Patients with HPI aged ≥65 years exhibited the highest risk (HR = 3.01, 95% CI = 1.90–5.30, *p* < 0.001), followed by patients aged 45–64 years (HR = 1.69, 95% CI = 1.26–2.90, *p* <0.01) and <45 years (HR = 1.49, 95% CI = 1.12–2.49, *p* < 0.01). Patients were most likely to develop SRMD 5 years or more after diagnosis of HPI (HR = 3.33, 95% CI = 1.97–5.89, *p* < 0.001). The increased risk of SRMD in male patients with HPI (HR = 2.73, 95% CI = 1.53–4.79, *p* < 0.001) was greater than in female patients (HR = 1.14, 95% CI = 1.04–1.65, *p* < 0.05).

**Conclusion:**

Patients with HPI were associated with an increased risk for SRMD, with a higher risk in men, aged ≥65 years, and diagnosed for more than 5 years.

## Introduction

The dopaminergic system is involved in sleep-related movement disorders (SRMD) (1). Marked improvement was seen with dopamine agonists in patients with restless legs syndrome (RLS), who present an urge to move legs that worsens at night (2, 3). RLS-like symptoms produced with drugs that block the dopaminergic system also implicate the role of dopamine in RLS (4). The other most common SRMD is periodic limb movement disorder (PLMD), defined as leg movements seen by polysomnography and last 0.5–10 s occurring every 5–90 s (5). In patients with RLS or PLMD, a decreased number of striatal D2-receptors demonstrated by SPECT imaging, in addition to pharmacological evidence, suggests dopaminergic dysfunction (6).

Evidence increasingly suggests that *Helicobacter pylori* infection (HPI) is associated with Parkinson's disease (PD). Chronic HPI may predispose people to idiopathic PD, and a higher prevalence of HPI has been reported in patients with PD (7, 8). HPI affects levodopa absorption, and its eradication was approved to improve clinical response to levodopa (9). Screening and eradication of HPI have been recommended, particularly for PD patients with erratic response to levodopa (10). HPI may alter levodopa absorption, reducing the effectiveness of the medication for PD, which may also affect the response to dopaminergic agents for patients with SRMD. Additionally, inflammatory factors such as IL-6 and TNF-alpha, induced by chronic infection such as HPI, can lead to the destruction of dopaminergic neurons (11, 12). Therefore, it has been proposed that gut microflora, including *Helicobacter pylori* may produce metabolites, trigger neuroinflammation, and affect the dopaminergic system by crossing the gut-brain axis (13, 14). Notably, in a mouse model of HPI, women with HPI-associated chronic gastritis had lower activity and forelimb lift counts, as well as prolonged sleep latency and shortened sleep duration (15).

The prevalence of HPI in Taiwan was 53.9%, and it remains high in most developing countries (16). A recent prospective study suggested that HPI was related to the occurrence of RLS (17), but patients with other SRMD were not included. A large-scale longitudinal study is essential for providing epidemiological evidence of the relationship between HPI and SRMD before effective management, such as HPI eradication, is recommended. This is the first study investigating the association between HPI with both RLS and PLMD. It aims to ascertain whether patients with HPI have an elevated risk of developing SRMD during more than 5 years of follow-up, using data from the Taiwan National Health Insurance Research Database (NHIRD).

## Materials and methods

### Data source

The study was conducted using the data deduced from the Longitudinal Health Insurance Database (LHID), which is a sub-database of the Taiwan National Health Insurance (NHI) program covering over 99% of 23 million citizens. The LHID contains records on inpatients, outpatients, and ambulatory care services from 2000 to 2013. The diagnostic coding system was the International Classification of Diseases, Ninth Revision, Clinical Modification (ICD-9-CM). The data have been de-identified before releasing for research.

### Study sample

**Figure 1:**
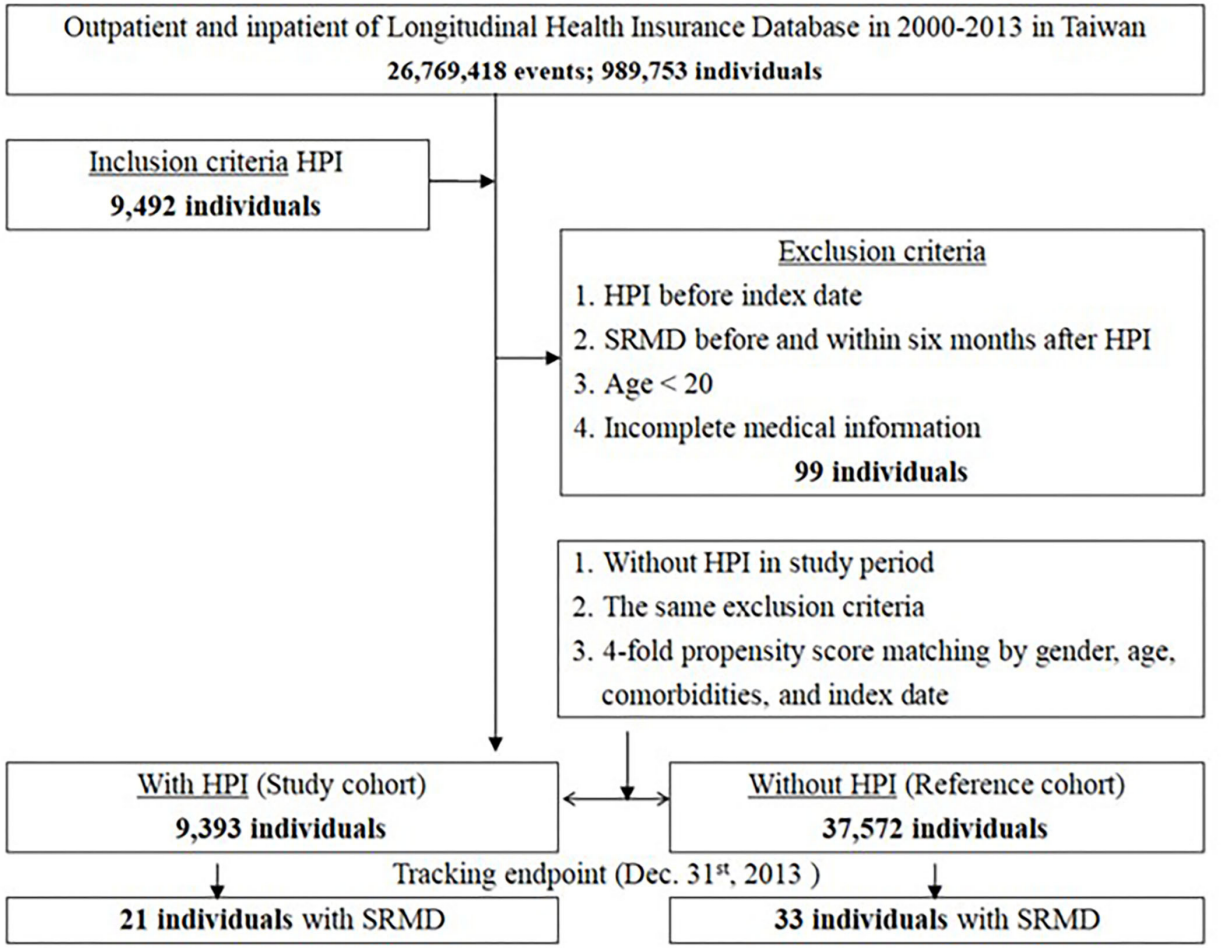
The flowchart of study sample selection from The National Health Insurance Research Database in Taiwan.

The study group and a comparison reference cohort were selected from the LHID (Figure 1). The study group comprised all patients who had been diagnosed with HPI based on ICD-9-CM code 041.86 for the first time from 2000 to 2013 (*N* = 9,393). In Taiwan, HPI is diagnosed during endoscopy by the rapid urease test or histologic analysis. HPI is usually treated with triple eradication therapy (amoxicillin/metronidazole, levofloxacin/clarithromycin, and a proton pump inhibitor) for at least 2 weeks to help prevent the bacteria from developing resistance to one particular antibiotic. Patients aged under 20 years and patients with a diagnosis of SRMD before or within 6 months after confirmation of HPI were excluded. The index date of the HPI cohort was the first date of diagnosis of HPI. We randomly selected 37,572 subjects (a sample size 4-fold that of the HPI group), and the index date of these age- and sex-matched comparison cohorts was randomly assigned a month and a day in the same year as the matched cases. Each patient was followed up from the index date until the diagnosis date of SRMD. For those who did not have SRMD, the last day of follow-up was defined as the date of insurance withdrawal or the last day of the study period (31 December 2013).

### Definitions of SRMD by ICD classification

The diagnosis of SRMD was based on ICD-9-CM codes 327.5 (PLMD) and 333.9 (RLS). In Taiwan, the diagnosis of SRMD was made by board-certified neurologists. The diagnosis of RLS was made according to the consensus clinical features of RLS as delineated by the International Restless Legs Syndrome Study Group (IRLSSG) (18, 19). The diagnosis of PLMD was made based on the diagnostic criteria in the International Classification of Sleep Disorders (5). The SRMD-associated comorbidity was defined as the individual with a history of the comorbidity before the index date, and it included diabetes mellitus (DM, ICD-9-CM 250), iron deficiency anemia (IDA, ICD-9-CM 280), depression (ICD-9-CM 296.2, 296.3, 300.4, and 311), anxiety (ICD-9-CM 300.00), sleep disorder (ICD-9-CM 307.4, 780.5), Parkinson's disease (ICD-9-CM 332), and renal disease (ICD-9-CM 580-589).

### Statistical analysis

Continuous variables were presented as mean and standard deviation (SD) and categorical variables as numbers and percentages. Differences between study and comparison cohorts in the distribution of demographic characteristics and comorbidities were examined by *t*-test (for age) and chi-square test (for sex and comorbidities). The incidence density of SRMD was measured for HPI and comparison cohorts, and the total SRMD events were divided by the total sum of follow-up years (per 1,000 person-years). Cox proportional hazard regression analysis was performed to calculate adjusted hazard ratios (HR), with 95% confidence intervals (CI), for SRMD risk between the two cohorts. To investigate the interaction of covariates in relation to the association between HPI and SRMD, we calculated adjusted HR stratified by age (< 45, 45–64, and ≥65 years), sex, and follow-up time. We also measured and compared the cumulative incidence curves between HPI and comparison cohorts by the Kaplan-Meier method and tested the curve differences by the log-rank test. All statistical analyses were performed using the SPSS software version 22.0. A 2-tailed *P*-value of less than 0.05 was considered statistically significant.

## Results

A total of 9,393 patients diagnosed with HPI and 37,572 age- and sex-matched controls for reference were included in this cohort study. The demographic characteristics of both groups are presented in Table 1. There were no significant differences in the distribution of age, sex, and comorbidities between the study group with HPI and the comparison cohort. The mean age of both cases and their controls was about 51 years on the index date, and most participants in the two cohorts were men (55.37%).

**Table 1:** Baseline demographic status and comorbidity compared between HPI and comparison groups.

**Variables**	**HPI cohort** ***N* = 9,393 (%)**	**Comparison cohort** ***N* = 37,572 (%)**	***P-*** **value**
Age, years (SD)^*^	51.88 ± 15.78	51.63 ± 16.44	0.999
<45	2,307 (24.56)	9,228 (24.56)	
45–64	3,375 (35.93)	13,500 (35.93)	
≧65	3,711 (39.51)	14,844 (39.51)	
**Sex**		0.999	
Male	5,201 (55.37)	20,804 (55.37)	
Female	4,192 (44.63)	16,768 (44.63)	
**Comorbidity**			
DM	1,287 (13.70)	4,897 (13.03)	0.087
IDA	321 (3.42)	1,345 (3.58)	0.447
Depression	303 (3.23)	1,240 (3.30)	0.717
Anxiety	689 (7.34)	2,711 (7.22)	0.689
Sleep disorder	224 (2.38)	881 (2.34)	0.819
Parkinson's disease	30 (0.32)	120(0.32)	0.999
Renal disease	396 (4.22)	1,511(4.02)	0.393

*DM*, diabetes mellitus; *IDA*, iron deficiency anemia; *HPI, Helicobacter pylori* infection.

* *t*-test.

Our data showed that over a 10-year follow-up on average, 21 patients with HPI developed SRMD with an overall rate of 0.43 cases per 1,000 person-years, of which 4 were RLS and 17 were PLMD. In the comparison cohort, 33 patients without HPI developed SRMD with an overall rate of 0.18 cases per 1,000 person-years, and 2 and 31 were RLS and PLMD, respectively. The results revealed that patients with HPI had a 2.18 times (95% CI = 1.26–3.82, *p* < 0.01) higher risk of developing SRMD compared to individuals without HPI (Table 2). To explore whether HPI is an age-dependent risk factor for SRMD, patients were divided into 3 groups, namely, <45, 45–64, and ≥65 years. The results showed that in comparison with age/sex matched controls, HPI patients aged ≥65 years exhibited the highest risk of developing SRMD (HR = 3.01, 95% CI = 1.90–5.30, *p* < 0.001), followed by patients aged 45–64 years (HR = 1.69, 95% CI = 1.26–2.90, *p* < 0.01), and <45 years (HR = 1.49, 95% CI = 1.12–2.49, *p* < 0.01). We also examined if HPI is a sex-dependent risk factor for SRMD. The Cox regression analysis revealed that the increased risk of SRMD in male patients with HPI (HR = 2.73, 95% CI = 1.53–4.79, *p* < 0.001) was greater than in female patients (HR = 1.14, 95% CI = 1.04–1.65, *p* < 0.05).

**Table 2:** Factors of SRMD subgroup stratified by age group or gender for Cox regression analysis.

	**HPI cohort**			**Comparison cohort**			**Crude HR (95%CI)**	**Adjusted HR (95%CI)**
**Variables**	**Events**	**PYs**	**Rate**	**Events**	**PYs**	**Rate**		
All-cause SRMD	21	49,084.02	0.43	33	182,636.26	0.18	2.29 (1.47–3.97)^**^	2.18 (1.26–3.82)^**^
RLS	4	49,084.02	0.08	2	182,636.26	0.01	7.19 (4.61–12.49)^***^	6.85 (3.99–45.12)^***^
PLMD	17	49,084.02	0.35	31	182,636.26	0.17	1.97 (1.26–3.43)^**^	1.88 (1.09–3.63)^*^
<45 years								
All-cause SRMD	3	9,298.77	0.32	7	33,382.48	0.21	1.49 (1.05–2.58)^**^	1.49 (1.12–2.49)^**^
RLS	2	9,298.77	0.22	0	33,382.48	0.00	∞	∞
PLMD	1	9,298.77	0.11	7	33,382.48	0.21	1.10 (0.32–2.86)	1.02 (0.37–3.17)
45–64 years								
All–cause SRMD	6	15,072.65	0.40	12	51,920.04	0.23	1.67 (1.25–2.86)^**^	1.69 (1.26–2.90)^**^
RLS	1	15,072.65	0.07	2	51,920.04	0.04	2.00 (1.30–4.66)^**^	2.10 (1.30–4.68)^**^
PLMD	5	15,072.65	0.33	10	51,920.04	0.19	1.52 (1.07–2.70)^**^	1.59 (1.15–2.79)^*^
≧65 years								
All–cause SRMD	12	24,712.60	0.49	14	97,333.73	0.14	3.27 (2.09–5.67)^***^	3.01 (1.90–5.30)^***^
RLS	1	24,712.60	0.04	0	97,333.73	0.00	∞	∞
PLMD	11	24,712.60	0.45	14	97,333.73	0.14	2.99 (1.91–5.19)^***^	2.85 (1.65–19.15)^***^
Male								
All–cause SRMD	18	28,446.99	0.63	21	98,616.03	0.21	2.87 (1.84–4.99)^***^	2.73 (1.53–4.79)^***^
RLS	4	28,446.99	0.14	2	98,616.03	0.02	6.70 (4.29–11.63)^***^	6.38 (3.70–42.91)^***^
PLMD	14	28,446.99	0.49	19	98,616.03	0.19	2.47 (1.58–4.29)^***^	2.35 (1.36–4.18)^***^
Female								
All–cause SRMD	3	20,637.03	0.15	12	84,020.22	0.14	0.99 (0.63–1.78)	1.14 (1.04–1.65)^*^
RLS	0	20,637.03	0.00	0	84,020.22	0.00	–	–
PLMD	3	20,637.03	0.15	12	84,020.22	0.14	0.99 (0.63–1.78)	1.14 (1.04–1.65)^*^

Model adjusted for age, sex, DM, IDA, depression, anxiety, sleep disorder, Parkinson's disease, and renal disease.

*Adjusted HR*, adjusted hazard ratio: adjusted for the variables listed in Table 1; *CI*, confidence interval; *DM*, diabetes mellitus; *IDA*, iron deficiency anemia; *PLMD*, periodic limb movement disorder; *Pys*, person-years; rate, incidence rate, per 1,000 person-years; *RLS*, restless legs syndrome; *SRMD*, sleep-related movement disorders.

* *P* < 0.05.

** *P* < 0.01.

*** *P* < 0.001.

**Figure 2:**
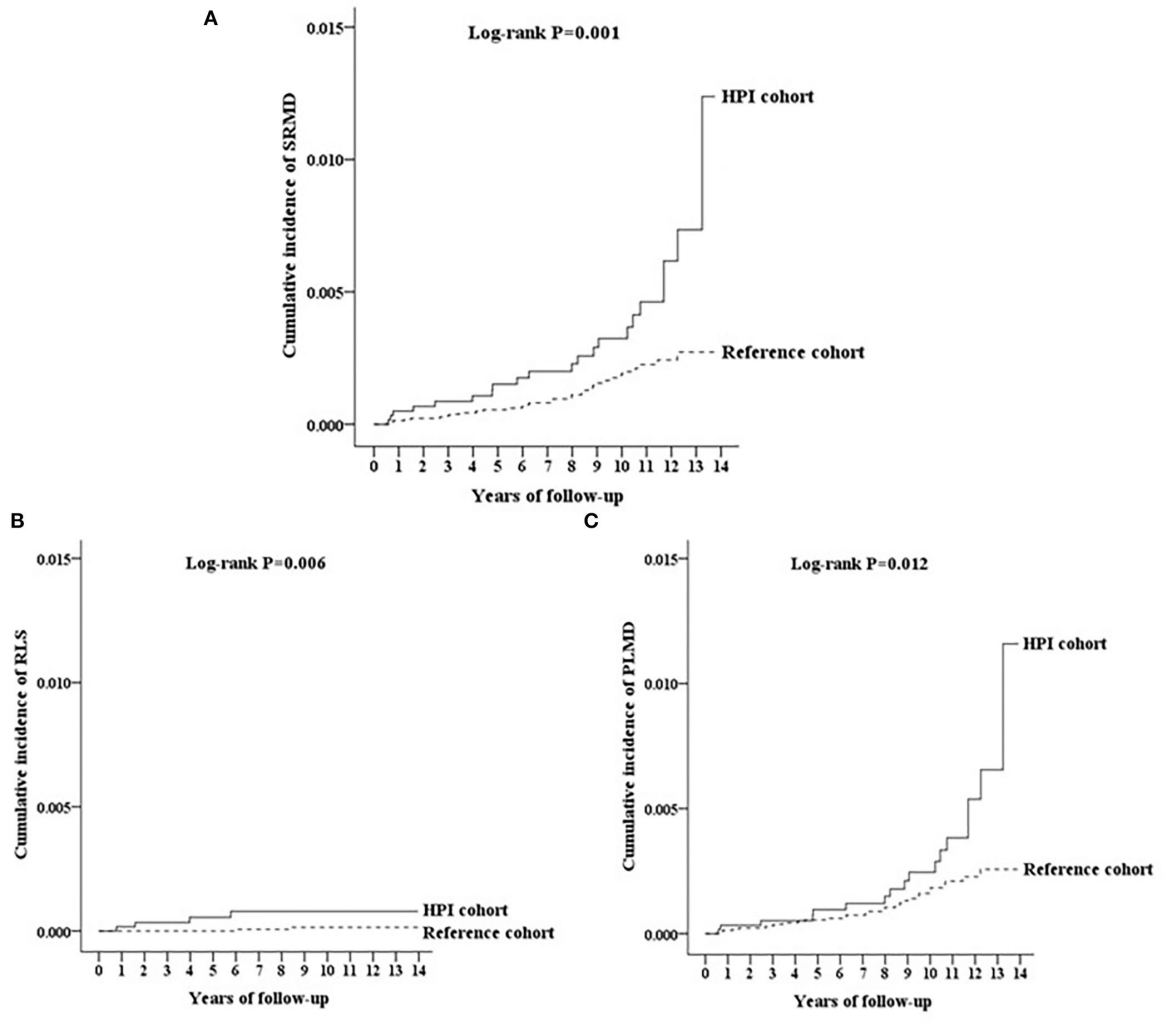
**(A)** The cumulative incidence curves of sleep-related movement disorders (SRMD) for individuals with and without *Helicobacter pylori* infection (HPI). **(B)** The cumulative incidence curves of restless legs syndrome (RLS) for individuals with and without HPI. **(C)** The cumulative incidence curves of periodic limb movement disorder (PLMD) for individuals with and without HPI.

We further analyzed the incidence of SRMD and SRMD subtypes using multivariate Cox proportional hazards regression analysis based on time intervals. Table 3 shows the impact of follow-up time on the risk of developing SRMD. Patients were most likely to develop SRMD after diagnosis of HPI for 5 years (SRMD, HR = 3.33, 95% CI = 1.97–5.89, *p* < 0.001; RLS, HR = 2.83, 95% CI = 1.68–4.93, *p* < 0.001; PLMD, HR = 3.39, 95% CI = 1.97–5.92, *p* < 0.001) (Table 3). Relatively, the risks for patients with HPI of developing SRMD were both about two times higher than controls in the groups with a follow-up time within 3 and between 3 and 5 years (HR = 2.18, 95% CI = 1.04–3.82, *p* < 0.05 and HR = 2.19, 95% CI = 1.27–3.83, *p* < 0.001, respectively) (Table 3). Kaplan–Meier analysis showed that, compared to the matched controls, patients with HPI had a significantly higher incidence of SRMD (log-rank test *p*= 0.001, Figure 2A), RLS (log-rank test *p*=0.006, Figure 2B), and PLMD (log-rank test *p* = 0.012, Figure 2C).

**Table 3:** Factors of SRMD subgroup stratified by follow-up time for Cox regression analysis.

	**HPI cohort**			**Comparison cohort**				
**Variables**	**Events**	**PYs**	**Rate**	**Events**	**PYs**	**Rate**	**Crude HR (95% CI)**	**Adjusted HR (95% CI)**
**Follow** **<3 years**								
SRMD	5	3,290.78	1.52	7	7,190.02	0.97	2.29 (1.07–3.98)^*^	2.18 (1.04–3.82)^*^
RLS	2	3,290.78	0.61	0	7,190.02	0.00	∞	∞
PLMD	3	3,290.78	0.91	7	7,190.02	0.97	1.38 (1.02–2.38)^*^	1.31 (1.01–2.29)^*^
**Follow** **≧3**, **<5 years**								
SRMD	3	5,042.06	0.59	4	10,524.72	0.38	2.30 (1.47–3.98)^***^	2.19 (1.27–3.83)^***^
RLS	1	5,042.06	0.20	0	10,524.72	0.00	∞	∞
PLMD	2	5,042.06	0.40	4	10,524.72	0.38	1.53 (1.10–2.66)^**^	1.46 (1.10–2.56)^**^
**Follow** **≧5 years**								
SRMD	13	40,751.18	0.32	22	164,921.51	0.13	3.51 (2.25–6.08)^***^	3.33 (1.97–5.89)^***^
RLS	1	40,751.18	0.02	2	164,921.51	0.01	2.97 (1.90–5.15)^***^	2.83 (1.68–4.93)^***^
PLMD	12	40,751.18	0.29	20	164,921.51	0.12	3.56 (2.28–6.18)^***^	3.39 (1.97–5.92)^***^

Model adjusted for age, sex, DM, IDA, depression, anxiety, sleep disorder, Parkinson's disease, and renal disease.

*Adjusted HR*, adjusted hazard ratio: adjusted for the variables listed in Table 1; *CI*, confidence interval; *DM*, diabetes mellitus; *IDA*, iron deficiency anemia; *PLMD*, periodic limb movement disorder; *Pys*, person-years; rate, incidence rate, per 1,000 person-years; *RLS*, restless legs syndrome; *SRMD*, sleep-related movement disorders.

* *P* < 0.05.

** *P* < 0.01.

*** *P* < 0.001.

## Discussion

In this nationwide population-based study, we found that patients with HPI had a significantly higher risk of developing subsequent SRMD. Patients with HPI aged ≥65 years, compared to the other age groups, were at the highest risk of developing RLS and PLMD, the most common types of SRMD. The increased risk of SRMD in male patients with HPI was greater than in female patients. Furthermore, patients were most likely to develop SRMD 5 years or more after diagnosis of HPI, suggesting that chronic infection due to an intestinal bacterial overgrowth is associated with the pathogenesis of SRMD.

The pathogenesis of RLS and other SRMD could be partially attributed to an immune reaction to gastrointestinal bacteria, leading to an immunological attack on the nervous system (20). Chronic neuroinflammation in the central nervous system may therefore result in iron deficiency-induced RLS (21). It has been reported that patients who lack ferritin light chain (FTL), an abnormality in iron metabolism, experienced atypical restless legs syndrome (22). HPI has been suggested to be involved in the pathogenesis of sleep disorders such as obstructive sleep apnea, due to inflammatory cytokines which result in neuropsychological impairment (23–25). Herein, we first provide evidence that HPI is associated with the development of subsequent SRMD.

This study demonstrated that patients with HPI had a 2.18 times (95% CI = 1.26–3.82) higher risk of developing SRMD compared to those without HPI. In comparison, a similarly high risk of PD in the HPI group (HR = 2.29, 95% CI = 1.44–3.66) was reported in a retrospective cohort study (26). We found that people of different ages are differentially influenced by HPI to develop subsequent SRMD, and patients with HPI aged ≥65 years exhibited the highest risk of developing SRMD (HR = 3.01, 95% CI = 1.90–5.30). Similarly, HPI was previously found to be significantly associated with an increased risk of PD among individuals aged 60 years and older (HR = 2.53, 95% CI = 1.47–4.35), but not among those younger than 60 years (26). Our age-stratified analysis suggests that the increased risk of RLS is more significant than PLMD among all age groups. But the numbers of RLS patients were too small to conduct further statistical analysis. In addition, our results showed that patients were most likely to develop SRMD, especially PLMD, after diagnosis of HPI for 5 years or more. In fact, chronic HPI has been demonstrated to induce the production of systemic pro-inflammatory cytokines that may cross the blood–brain barrier to cause a vicious cycle of uncontrolled neuroinflammation (27).

According to a recent meta-analysis, the odds ratio of PD for those with HPI was 1.59 (95% CI = 1.37–1.85), compared to individuals without HPI (8). In contrast, an earlier review reported that the prevalence of HPI in patients with PD in four studies ranged from 37% to 59%, which was similar to that of the general population (28). Moreover, only limited evidence could be provided in three randomized controlled trials (RCT) to support that HPI eradication alleviated motor symptoms in patients with PD (28). A wellconducted RCT with standard outcome measures and treatment is also required to demonstrate whether patients with HPI have a higher risk of developing subsequent SRMD.

Both RLS and PLMD have been demonstrated to be associated with an increased risk for stroke in our previous study (29). Chronic HPI has also been shown to increase the risk of acute ischemic stroke (OR = 2.57, 95% CI = 1.09–6.08) in the Fukuoka Harasanshin Atherosclerosis Trial (30). A future study will demonstrate whether patients with HPI have an even higher risk of stroke when they develop SRMD due to hypothetical neuroinflammation and dysfunction of the dopaminergic system and show if HPI eradication for patients with SRMD can reduce the risk of subsequent stroke.

In contrast with a prospective study of a smaller scale on the relationship between HPI and occurrence of RLS (17), the diagnosis of SRMD in this study was made based on standard criteria in our nationwide health care system, with the correctly collected time interval between diagnosis of HPI and subsequent SRMD (2, 29). This partially accounts for a relatively low prevalence of SRMD in our population, suggesting that the disease state of SRMD may be more advanced and detectable by polysomnography than those evaluated by primary screening instruments in other studies.

There are several limitations to this study. First, neither different severities of SRMD nor health information such as intensity of tobacco use, alcohol consumption, and diet that may influence the risk of SRMD and HPI could be provided by the database. Some potential risk factors for SRMD, such as genetic background and family history of SRMD, were not available. Second, HPI may or may not produce any clinical symptoms, and most endoscopy and tissue biopsy for HPI are performed when people report symptoms. Therefore, some patients may be underdiagnosed and recruited into the matched controls. Furthermore, we did not know if the patients were treated completely or declared cured of HPI. In fact, the recurrence of HPI is not rare, which can be caused by recrudescence and reinfection (31). Finally, the data may also include unidentified recurrent patients who had SRMD and HPI before 1996 when NHI was enacted (32). Nevertheless, we only included patients with new incidences of HPI and SRMD to increase the accuracy of the diagnoses. The criteria in this study depended on ICD codes, which may be different from other studies, and the NHI administration has a cross-checking system to continuously evaluate the precision of records (33).

## Conclusion

*Helicobacter pylori* infection was demonstrated to be associated with an increased risk of subsequent SRMD such as RLS and PLMD in this study. Patients with HPI aged ≥65 years exhibited the greatest risk of developing SRMD. Patients were most likely to develop SRMD after diagnosis of HPI for 5 years or more. It suggests that chronic inflammation after HPI is associated with the pathogenesis of SRMD, especially in the vulnerable group in which patients are older and potentially have an underlying neurodegenerative disease. Further research is required to explore whether effective practices, such as HPI eradication, can reduce the risk of developing SRMD.

## Data availability statement

The original contributions presented in the study are included in the article/supplementary material, further inquiries can be directed to the corresponding author.

## Ethics statement

This study protocol was reviewed by Institutional Review Board of TriService General Hospital (TSGHIRB No.: 2-104-05-045). Written informed consent from the patients/participants or patients/participants' legal guardian/next of kin was not required to participate in this study in accordance with the national legislation and the institutional requirements.

## Author contributions

Y-FS, J-HY, K-HL, C-HCho, J-TL, C-LT, C-HChu, and W-CC contributed substantially to the research concept and design, acquisition, analysis, and interpretation of data. C-HCho, K-HL, Y-KL, S-YC, and Y-FS drafted and revised the manuscript to be published. All authors contributed to the article and approved the submitted version.

## Funding

This study was supported by grants from the Ministry of Science and Technology Taiwan (MOST-109-2314-B-016-008), the Ministry of National Defense Medical Affairs Bureau (MND-MAB-C06-112025, MNDMAB-C-111-07-111028, and MND-MAB-C07-111028), and Tri-Service General Hospital (TSGH-E-111228, TSGH-E-110196, TSGH-E-109226, and TSGH-C108-006-007-007-S05). The funders had no role in study design, data collection and analysis, decision to publish, or preparation of the manuscript.

## Conflict of interest

The authors declare that the research was conducted in the absence of any commercial or financial relationships that could be construed as a potential conflict of interest.

## Publisher's note

All claims expressed in this article are solely those of the authors and do not necessarily represent those of their affiliated organizations, or those of the publisher, the editors and the reviewers. Any product that may be evaluated in this article, or claim that may be made by its manufacturer, is not guaranteed or endorsed by the publisher.

## References

[1] Ferini-Strambi L Carli G Casoni F Galbiati A. Restless legs syndrome and parkinson disease: a causal relationship between the two disorders? Front Neurol. (2018) 9:551. 10.3389/fneur.2018.00551 30087647 PMC6066514

[2] Allen RP Picchietti DL Garcia-Borreguero D. Restless legs syndrome/Willis-Ekbom disease diagnostic criteria: updated international restless legs syndrome study group (IRLSSG) consensus criteria–history, rationale, description, and significance. Sleep Med. (2014) 15:860–73. 10.1016/j.sleep.2014.03.025 25023924

[3] Tergau F Wischer S Wolf C Paulus W. Treatment of restless legs syndrome with the dopamine agonist alpha-dihydroergocryptine. Mov Disord. (2001) 16:731–5. 10.1002/mds.1141 11481700

[4] Winkelmann J Schadrack J Wetter TC Zieglgansberger W Trenkwalder C. Opioid and dopamine antagonist drug challenges in untreated restless legs syndrome patients. Sleep Med. (2001) 2:57–61. 10.1016/S1389-9457(00)00025-3 11152983

[5] Littner MR Kushida C Anderson WM. Practice parameters for the dopaminergic treatment of restless legs syndrome and periodic limb movement disorder. Sleep. (2004) 27:557–9. 10.1093/sleep/27.3.557 15164914

[6] Michaud M Soucy JP Chabli A Lavigne G Montplaisir J, SPECT SPECT imaging of striatal pre- and postsynaptic dopaminergic status in restless legs syndrome with periodic leg movements in sleep. J Neurol. (2002) 249:164–70. 10.1007/PL00007859 11985381

[7] Dobbs RJ Charlett A Dobbs SM Weller C Peterson DW. Parkinsonism: differential age-trend in *Helicobacter pylori* antibody. Aliment Pharmacol Ther. (2000) 14:1199–205. 10.1046/j.1365-2036.2000.00815.x 10971237

[8] Shen X Yang H Wu Y Zhang D Jiang H. Meta-analysis: association of *Helicobacter pylori* infection with Parkinson's diseases. Helicobacter. (2017) 22:e12398. 10.1111/hel.12398 28598012

[9] Hashim H Azmin S Razlan H. Eradication *of Helicobacter pylori* infection improves levodopa action, clinical symptoms and quality of life in patients with Parkinson's disease. PLoS ONE. (2014) 9:e112330. 10.1371/journal.pone.0112330 25411976 PMC4239049

[10] Liu H Su W Li S. Eradication of *Helicobacter pylori* infection might improve clinical status of patients with Parkinson's disease, especially on bradykinesia. Clin Neurol Neurosurg. (2017) 160:101–4. 10.1016/j.clineuro.2017.07.003 28704778

[11] Dobbs RJ Charlett A Purkiss AG Dobbs SM Weller C Peterson DW. Association of circulating TNF-alpha and IL-6 with ageing and parkinsonism. Acta Neurol Scand. (1999) 100:34–41. 10.1111/j.1600-0404.1999.tb00721.x 10416510

[12] Wang XM Zhang YG Li AL. Relationship between levels of inflammatory cytokines in the peripheral blood and the severity of depression and anxiety in patients with Parkinson's disease. Eur Rev Med Pharmacol Sci. (2016) 20:3853–6. 27735031

[13] McGee DJ Lu XH Disbrow EA. Stomaching the possibility of a pathogenic role for *Helicobacter pylori* in Parkinson's disease. J Parkinsons Dis. (2018) 8:367–74. 10.3233/JPD-181327 29966206 PMC6130334

[14] Gorle N Bauwens E Haesebrouck F Smet A Vandenbroucke RE. Helicobacter and the potential role in neurological disorders: there is more than *Helicobacter pylori*. Front Immunol. (2020) 11:584165. 10.3389/fimmu.2020.584165 33633723 PMC7901999

[15] Liu H Zheng W Zhang L Lin T Tang Y Hu L. Effect of *Helicobacter pylori*-associated chronic gastritis on autonomous activity and sleep quality in mice. Front Pharmacol. (2022) 13:785105. 10.3389/fphar.2022.785105 35185560 PMC8856107

[16] Hooi JKY Lai WY Ng WK. global prevalence of *Helicobacter pylori* infection: systematic review and meta-analysis. Gastroenterology. (2017) 153:420–9. 10.1053/j.gastro.2017.04.022 28456631

[17] Rezvani F Sayadnasiri M Rezaei O. Restless legs syndrome in patients infected with *Helicobacter pylori*. Neurol Res. (2018) 40:581–5. 10.1080/01616412.2018.1454704 29623817

[18] Walters AS. Toward a better definition of the restless legs syndrome. Int Restless Legs Synd Study Group Mov Disord. (1995) 10:634–42. 10.1002/mds.870100517 8552117

[19] Zucconi M Ferri R Allen R. The official world association of sleep medicine (WASM) standards for recording and scoring periodic leg movements in sleep (PLMS) and wakefulness (PLMW) developed in collaboration with a task force from the international restless legs syndrome study group (IRLSSG). Sleep Med. (2006) 7:175–83. 10.1016/j.sleep.2006.01.001 16459136

[20] Weinstock LB Walters AS Paueksakon P. Restless legs syndrome—theoretical roles of inflammatory and immune mechanisms. Sleep Med Rev. (2012) 16:341–54. 10.1016/j.smrv.2011.09.003 22258033

[21] Hamed SA. Neurologic conditions and disorders of uremic syndrome of chronic kidney disease: presentations, causes, and treatment strategies. Expert Rev Clin Pharmacol. (2019) 12:61–90. 10.1080/17512433.2019.1555468 30501441

[22] Cozzi A Santambrogio P Privitera D. Human L-ferritin deficiency is characterized by idiopathic generalized seizures and atypical restless leg syndrome. J Exp Med. (2013) 210:1779–91. 10.1084/jem.20130315 23940258 PMC3754865

[23] Kountouras C Polyzos SA Stergiopoulos C. A potential impact of *Helicobacter pylori* infection on both obstructive sleep apnea and atrial fibrillation-related stroke. Sleep Med. (2017) 34:256. 10.1016/j.sleep.2017.03.010 28434882

[24] Kountouras J Polyzos SA Deretzi G. *Helicobacter pylori* associated with obstructive sleep apnea might contribute to sleep, cognition, and driving performance disturbances in patients with cirrhosis. Clin Gastroenterol Hepatol. (2015) 13:1547. 10.1016/j.cgh.2015.02.009 25697625

[25] Stergiopoulos C Kountouras J Daskalopoulou-Vlachoyianni E. *Helicobacter pylori* may play a role in both obstructive sleep apnea and metabolic syndrome. Sleep Med. (2012) 13:212–3. 10.1016/j.sleep.2011.04.016 22137108

[26] Huang HK Wang JH Lei WY Chen CL Chang CY Liou LS. *Helicobacter pylori* infection is associated with an increased risk of Parkinson's disease: a population-based retrospective cohort study. Parkinsonism Relat Disord. (2018) 47:26–31. 10.1016/j.parkreldis.2017.11.331 29174171

[27] Park AM Omura S Fujita M Sato F Tsunoda I. *Helicobacter pylori* and gut microbiota in multiple sclerosis versus Alzheimer's disease: 10 pitfalls of microbiome studies. Clin Exp Neuroimmunol. (2017) 8:215–32. 10.1111/cen3.12401 29158778 PMC5693366

[28] Rees K Stowe R Patel S. *Helicobacter pylori* eradication for Parkinson's disease. Cochrane Database Syst Rev. (2011) 2011:CD008453. 10.1002/14651858.CD008453.pub2 22071847 PMC13126632

[29] Chou CH Yin JH Chen SY. The potential impact of sleep-related movement disorders on stroke risk: a population-based longitudinal study. QJM. (2017) 110:649–55. 10.1093/qjmed/hcx097 28482057 PMC5914305

[30] Sawayama Y Ariyama I Hamada M. Association between chronic *Helicobacter pylori* infection and acute ischemic stroke: fukuoka harasanshin atherosclerosis Trial (FHAT). Atherosclerosis. (2005) 178:303–9. 10.1016/j.atherosclerosis.2004.08.025 15694938

[31] Sjomina O Pavlova J Niv Y Leja M. Epidemiology of *Helicobacter pylori* infection. Helicobacter. (2018) 23 Suppl 1:e12514. 10.1111/hel.12514 30203587

[32] Cheng TM. Reflections on the 20th anniversary of Taiwan's single-payer national health insurance system. Health Aff. (2015) 34:502–10. 10.1377/hlthaff.2014.1332 25732502

[33] Huang SK Tsai SL Hsu MT. Ensuring the sustainability of the Taiwan national health insurance. J Formos Med Assoc. (2014) 113:1–2. 10.1016/j.jfma.2013.08.010 24060196

